# Novel Multiplexed Assay for Identifying SH2 Domain Antagonists of STAT Family Proteins

**DOI:** 10.1371/journal.pone.0071646

**Published:** 2013-08-16

**Authors:** Kazuyuki Takakuma, Naohisa Ogo, Yutaka Uehara, Susumu Takahashi, Nao Miyoshi, Akira Asai

**Affiliations:** Center for Drug Discovery, Graduate School of Pharmaceutical Sciences, University of Shizuoka, Suruga-ku, Shizuoka, Japan; Emory University, United States of America

## Abstract

Some of the signal transducer and activator of transcription (STAT) family members are constitutively activated in a wide variety of human tumors. The activity of STAT depends on their Src homology 2 (SH2) domain-mediated binding to sequences containing phosphorylated tyrosine. Thus, antagonizing this binding is a feasible approach to inhibiting STAT activation. We have developed a novel multiplexed assay for STAT3- and STAT5b-SH2 binding, based on amplified luminescent proximity homogeneous assay (Alpha) technology. AlphaLISA and AlphaScreen beads were combined in a single-well assay, which allowed the binding of STAT3- and STAT5b-SH2 to phosphotyrosine peptides to be simultaneously monitored. Biotin-labeled recombinant human STAT proteins were obtained as N- and C-terminal deletion mutants. The spacer length of the DIG-labeled peptide, the reaction time, and the concentration of sodium chloride were optimized to establish a HTS system with Z’ values of greater than 0.6 for both STAT3- and STAT5b-SH2 binding. We performed a HTS campaign for chemical libraries using this multiplexed assay and identified hit compounds. A 2-chloro-1,4-naphthalenedione derivative, Compound **1**, preferentially inhibited STAT3-SH2 binding *in vitro*, and the nuclear translocation of STAT3 in HeLa cells. Initial structure activity relationship (SAR) studies using the multiplexed assay showed the 3-substituent effect on both the activity and selectivity of STAT3 and STAT5b inhibition. Therefore, this multiplexed assay is useful for not only searching for potential lead compounds but also obtaining SAR data for developing new STAT3/STAT5b inhibitors.

## Introduction

There is a growing need for technological developments of new high throughput screening (HTS) systems that promise fast and cost-effective performance in the drug discovery process. Multiplexing, where multiple targets or readouts are monitored in the same experiment, is a particularly useful technique for genetic analysis and drug discovery. Multiplexed screening in drug discovery has been reported for enzymes, nuclear receptors, and GPCRs so far [1]–[5]. However, there are no examples of multiplexed assay systems for identifying inhibitors of signal transducer and activator of transcription (STAT) family transcription factors. STAT family members were originally discovered as latent cytoplasmic transcription factors that transmit signals from cytokine receptors and growth factor receptors to the nucleus [6]. These signaling pathways involve the activation of receptor tyrosine kinases, such as epidermal growth factor and platelet-derived growth factor receptors, and Janus kinases (JAKs). Following phosphorylation at a conserved tyrosine residue, two STAT monomers dimerize through a reciprocal interaction between phosphotyrosine (pTyr, pY) and the Src homology 2 (SH2) domain. The STAT dimers subsequently translocate to the nucleus, where they regulate gene expression by binding to specific DNA sequences. The STAT family consists of seven members: STAT1-STAT4, STAT6, and the isoforms of STAT5, STAT5a and STAT5b. The members play a role in diverse biological functions, including cell proliferation, cell survival, angiogenesis, apoptosis, and inflammation [6]–[11].

STAT3 is constitutively activated in many types of hematopoietic and solid tumors, such as leukemia, breast cancer, and prostate cancer. Because of its central role just downstream of protein tyrosine kinases, aberrant STAT3 activity is often associated with transformation mechanisms induced by oncogenic tyrosine kinases. In addition, STAT3 is constitutively activated both in tumor cells and in immune cells confined in tumor microenvironments, and STAT3 inhibits the expression of mediators necessary for mounting an immune response against the tumor cells [12], [13]. STAT5b is also activated in several kinds of leukemias and solid tumors [11]. STAT5b was reported to potentiate v-Src-mediated transformation of NIH-3T3 cells [14]. The growth of squamous cell carcinoma of the head and neck cells was inhibited by antisense oligonucleotides for STAT5b [15], [16]. Similarly, in a mouse xenograft model, a dominant negative STAT5b mutant slowed the growth of prostate cancer cells [17]. These data demonstrate that both STAT3 and STAT5b are important therapeutic targets for anti-cancer chemotherapy.

The JAK family is composed from four different non-receptor tyrosine kinases, JAK1, JAK2, JAK3 and TYK2. JAK2 is activated by various cytokines and growth factors. JAK2 activation induces the phosphorylation of STAT3 and STAT5, which leads to their dimerization [18]. Several small molecule inhibitors of JAK2 have been reported [19]. Of the JAK2 inhibitors, Pyridone 6, also called JAK inhibitor 1, is an ATP-competitive pan-JAKs inhibitor [20]. It downregulates STAT3 activity and inhibits cell growth [21]. In addition, peptide-based STAT3 inhibitors designed to target the STAT3-SH2 domain were effective in suppressing the cellular functions of STAT3 [22], [23]. Inhibiting the dimerization of the STAT proteins through their SH2 domains is a unique mode of action, which is particularly desirable for developing new cancer therapies. Several small molecules, such as Stattic, S3I-201 and STA-21, inhibit STAT3 by targeting the SH2 domain [24]–[26]. Amplified luminescent proximity homogeneous assay (Alpha) technology can be used to analyze protein-protein or protein-peptide interactions [5], [27]–[29]. We have previously screened STAT3-SH2 binding inhibitors using a technique based on Alpha technology, and 5,15-diphenylporphyrin was identified as a selective STAT3-SH2 inhibitor [29]. However, only a few small molecule inhibitors have been reported for STAT5b-SH2 binding. One compound inhibited STAT5 tyrosine phosphorylation in Daudi cells and also decreased the amount of the STAT5/DNA complex in K562 cells [30].

In this study, to identify an SH2 antagonist chemotype screen for STAT3 and STAT5b, we developed a multiplexed assay of STAT3- and STAT5b-SH2 binding by combining AlphaLISA and AlphaScreen beads, which produce different colors, in a single-well assay. We conducted a HTS campaign of our in-house chemical libraries using this multiplex assay, and identified a cell permeable small molecule that preferentially antagonizes STAT3-SH2 binding. Development of the multiplexed assay, hit identification and initial structure activity relationship (SAR) studies are reported.

## Results and Discussion

### Design of the Multiplexed STAT3- and STAT5b-SH2 Binding Assay

**Figure 1:**
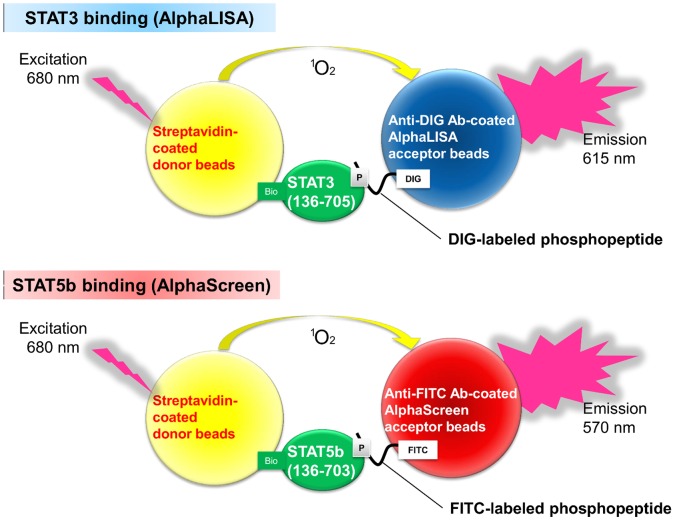
Schematic diagram of the multiplexed Alpha assay for STAT3- and STAT5-SH2 binding.

Previous studies have shown that Alpha technology can analyze protein-protein or protein-peptide interactions; therefore, we chose an Alpha assay system to detect the interaction between the SH2 domain and the peptide containing pTyr [31], [32]. We developed a multiplexed STAT3- and STAT5b-SH2 binding assay in a single well by combining AlphaLISA and AlphaScreen beads (Figure 1). Streptavidin-coated donor beads and antibody-conjugated acceptor beads were used in the Alpha assay. The AlphaLISA and AlphaScreen acceptor beads emitted their respective signals only when they were in close proximity to the donor beads, which meant that the labeled phosphorylated peptide and the biotinylated STAT protein were bound. The peptide sequence used for the STAT3 ligand, GpYLPQTV, was based on the sequence derived from the gp130 receptor, which has a high affinity for the STAT3 SH2 domain [33]. The peptide sequence used for the STAT5b ligand, GpYLVLDKW, was based on a sequence derived from the erythropoietin receptor, which has a high affinity for the STAT5b SH2 domain [34], [35]. These peptides have been used in previous studies [29], [33], [36], [37]. Therefore, the STAT3 binding was detected by the DIG-labeled GpYLPQTV peptide, and the biotinylated STAT3 protein was detected by the AlphaLISA system. The STAT5b binding was detected by the FITC-labeled GpYLVLDKW peptide, and the biotinylated STAT5b protein was detected by the AlphaScreen system. The multiplexed STAT3- and STAT5b-SH2 binding assay was conducted by measuring the two different signals simultaneously.

### Expression of Recombinant Human STAT3 and STAT5b Proteins

**Figure 2:**
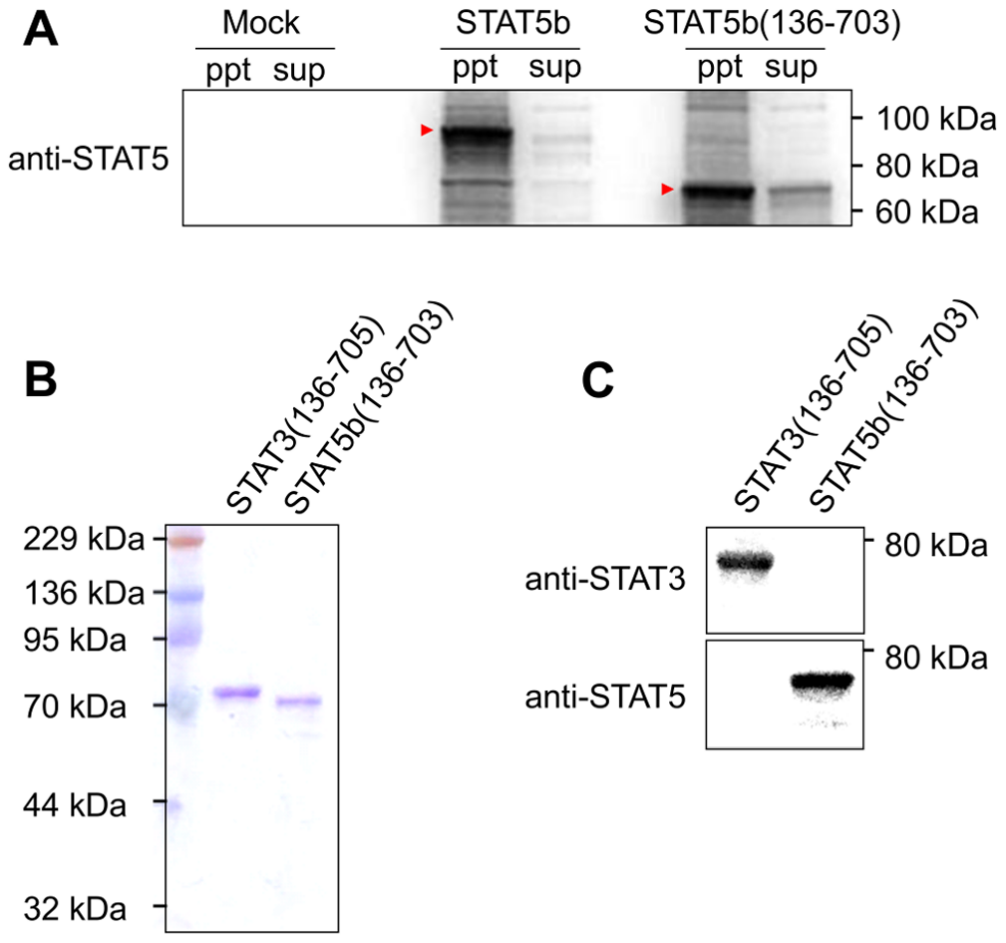
STAT3 and STAT5b protein expression. (A) Western blot analysis of the soluble (sup) and insoluble (ppt) fractions of *E. coli* expressing STAT5b.The proteins (5 µg) were analyzed by SDS-PAGE, and then probed with an antibody. The arrows indicate the expressed proteins. (B) CBB staining of the STAT3(136–705) and STAT5b(136–703) proteins. The purified soluble proteins (0.5 µg) were analyzed by SDS-PAGE. (C) Western blot analysis of the STAT3(136–705) and STAT5b(136–703) proteins. The blotted proteins were detected with an antibody.

We have previously prepared soluble recombinant human STAT3 and STAT1 proteins, which were suitable for the SH2 binding assay [29]. STAT5b was not expressed in the soluble fraction in *Escherichia coli* (Figure 2A). However, one of the truncated forms of STAT5b, STAT5b(136–703), in which the N- and C-terminal domains were deleted, was successfully expressed in the soluble fraction, although some of the protein remained in the insoluble fraction (Figure 2A). The truncated form of STAT3, STAT3(136–705), was also constructed. The amino acid sequence of STAT3(136–705) is homologous to that of STAT5b(136–703). The CBB staining and Western blotting analysis indicated that both the STAT3(136–705) and STAT5b(136–703) proteins were expressed as their soluble forms (Figures 2B, 2C). The Avi-tag biotinylation of both proteins was confirmed by immunoblotting with streptavidin-horseradish peroxidase conjugate (data not shown). Therefore, the truncated proteins, STAT3(136-705) and STAT5b(136-703), were used in the following studies.

### Development of the Multiplexed Binding Assay

**Figure 3:**
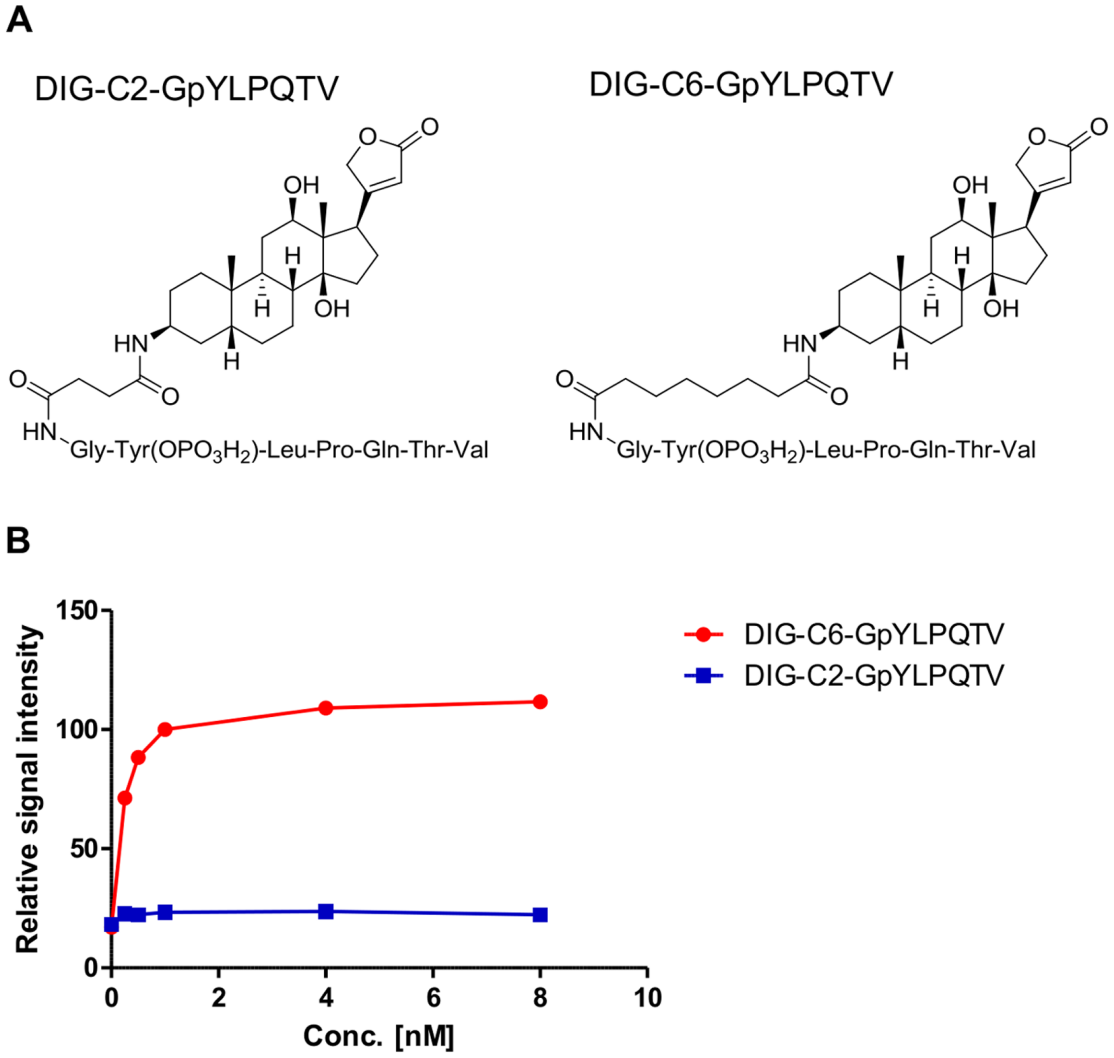
Effects of the spacer length in digoxygenin (DIG)-labeled GpYLPQTV peptides on STAT3 binding. (A) Chemical structures of the peptides. DIG-labeled peptides that contain two carbon (C2) or six carbon (C6) spacers were used. (B) Dose dependence of the DIG-C2-GpYLPQTV and DIG-C6-GpYLPQTV peptides on STAT3 binding. Each point represents the mean from three replicates, and the error bars represent the standard deviation from the mean. The signals from 1.0 nM DIG-C6-GpYLPQTV were used as 100%.

To acquire the signal window, the length of the spacer between DIG and the peptide sequence in the DIG-labeled GpYLPQTV peptide was investigated by using the single STAT3-SH2 binding assay. A six carbon (C6) and a two carbon (C2) peptide spacer were used (Figure 3A). The dose response studies demonstrated that the DIG-C6-GpYLPQTV peptide exhibited much higher signals than the DIG-C2-GpYLPQTV peptide, although the signals of both peptides increased in a peptide dose-dependent manner (Figure 3B). The spacer length of the labeled peptide had a significant effect on the signal window in this binding assay. The DIG-C6-GpYLPQTV peptide was chosen as the STAT3 ligand. When 100 nM STAT3(136–705) was used as the biotinylated protein, 2.0 nM DIG-C6-GpYLPQTV produced the maximum signal for the STAT3-SH2 binding (Figure 3B, Figure S1). For the STAT5b-SH2 binding, FITC-C6-GpYLVLDKW was used as the STAT5b ligand. The signals increased in a peptide dose-dependent manner (Figure S1). When 20 nM STAT5b(136–703) was used, 2.5 nM FITC-C6-GpYLVLDKW produced the maximum signal for STAT5b-SH2 binding. We also used the AlphaScreen system to demonstrate that DIG-C2-GpYLPQTV inhibited the binding between FITC-C6-GpYLPQTV and STAT3(136–705) (Figure S2). Therefore, although DIG-C2-GpYLPQTV could bind to STAT3, it was not suitable for the Alpha system. The anti-DIG antibody may access the DIG-C6-GpYLPQTV peptide-STAT3 protein complex in preference to the DIG-C2-GpYLPQTV peptide complex because of the length of the spacer.

To optimize the assay conditions, the stability to DMSO, the reaction time, and the effect of NaCl were examined for each STAT3- or STAT5b-SH2 binding assay. Because the test compounds are usually dissolved in DMSO, the assay systems must be robust to DMSO. The signals for 0.25%, 1.0%, and 4.0% DMSO were equal to those for 0% for both STAT3 and STAT5b (Figure S3). These results indicate that both the STAT3- and STAT5b-SH2 binding assays were stable to DMSO up to at least 4%. Next, we examined the stability for reaction times of 30, 60, 90, and 120 min, and the signals were similar for all reaction times (Figure S4). These results demonstrate that both STAT3- and STAT5b-SH2 binding assays were stable for reaction times of up to 120 min. Moreover, the signals were measured when 50, 100, and 200 mM NaCl were added to the reaction mixture. The signals for 100 mM and 200 mM NaCl were reduced to 31% and 11% for 50 mM NaCl, respectively, in 100 nM STAT3(136–705) for STAT3-SH2 binding (Figure S5). The signals decreased significantly for higher NaCl concentrations for both STAT3-SH2 and STAT5b-SH2 bindings. The interaction between the ligand containing pTyr and the SH2 domains is mainly mediated by hydrogen bonds between the negatively polarized oxygen atoms on the ligand phosphate group and the basic and polar amino acids in the SH2 domains [38]. These results suggest that the interactions between the peptides and the corresponding STAT proteins were mediated by the pTyr-SH2 domain. In both binding assays, the signals increased with the amount of protein and with the amount of peptide. When 1.0 nM DIG-C6-GpYLPQTV was used as a STAT3 ligand, 200 nM STAT3(136–705) produced the maximum signal for STAT3 binding. When 1.0 nM FITC-C6-GpYLVLDKW was used, 40 nM STAT5b(136–703) produced the maximum signal for STAT5b binding. Therefore, we chose 100 nM STAT3(136–705) and 1.0 nM DIG-C6-GpYLPQTV, and 20 nM STAT5b(136–703) and 1.0 nM FITC-C6-GpYLVLDKW were used for the following assays, because the signals were not saturated at these concentrations. The DMSO concentration was 1.0%, the reaction time was 90 min, and the NaCl concentration was 50 mM for both STAT3- and STAT5b-SH2 binding.

**Figure 4:**
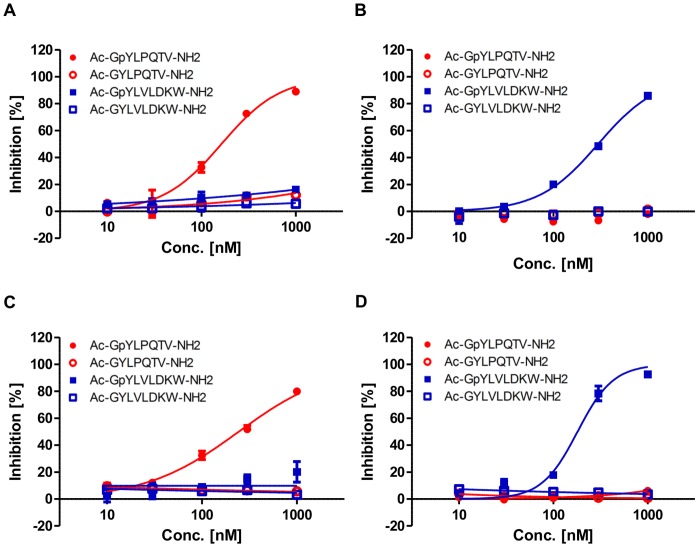
Inhibitory effects of the non-labeled peptides on STAT3- and STAT5b-SH2 binding. (A) Dose-dependent inhibition of STAT3 binding in the single assay. (B) Dose-dependent inhibition of STAT5b binding in the single assay. (C) Dose-dependent inhibition of STAT3 binding in the multiplexed assay. (D) Dose-dependent inhibition of STAT5b binding in the multiplexed assay. Each point represents the mean from three replicates, and the error bars represent the standard deviation from the mean.

To investigate the specificity of the interactions between the peptides and the corresponding STAT proteins, the inhibitory effect of phosphorylated or non-phosphorylated non-labeled peptides that contained a tyrosine residue was determined in each single binding assay. In the single STAT3-SH2 binding assay, the non-labeled peptide for STAT3, Ac-GpYLPQTV-NH_2_, inhibited the STAT3-SH2 binding, whereas the non-phosphorylated peptide, Ac-GYLPQTV-NH2, and the unrelated peptides, Ac-GpYLVLDKW-NH_2_ and Ac-GYLVLDKW-NH_2_, had no effect (Figure 4A). In contrast, the non-labeled peptide for STAT5b, Ac-GpYLVLDKW-NH_2_, inhibited STAT5b-SH2 binding, and the non-phosphorylated peptide, Ac-GYLVLDKW-NH_2_, and the unrelated peptides, Ac-GpYLPQTV-NH_2_ and Ac-GYLPQTV-NH_2_, had no effect in the single STAT5b-SH2 binding assay (Figure 4B). These results suggest that the binding is mediated by the interactions between the SH2 domain of the proteins and pTyr in the peptides. Furthermore, the inhibitory effect of the peptides in the multiplexed binding assay was similar to that in the single assays (Figures 4C, 4D). Moreover, the STAT3 small molecule inhibitor, Stattic, and the STAT5 inhibitor selectively inhibited the STAT3- and STAT5b-SH2 binding, respectively, in the multiplexed assays (Figure S6). The IC_50_ values of Stattic were 50 µM and 175 µM for the STAT3- and STAT5b-SH2 binding, respectively, and those of STAT5 inhibitor were 161 µM and 2.8 µM. These results confirm that our multiplexed assay is suitable for identifying selective STAT3 and STAT5b inhibitors. The Z’ value is a parameter calculated from both the assay signal dynamic range and the data variation, so the value that is over 0.5 is suitable for HTS [39]. The Z’ values in the multiplexed assay were greater than 0.6, which is compatible with HTS campaigns (Table 1). Therefore, STAT3- or STAT5b-SH2 binding inhibitors can be simultaneously screened using our multiplexed assay.

**Table 1:** Determination of the Z’ values by the multiplexed assay.

	Z’ values			
**STAT3 binding**	0.79	0.83	0.89	0.69
**STAT5b binding**	0.66	0.64	0.73	0.69

The Z’ values acquired from four independent experiments are shown.

Our multiplexed binding assay offers several advantages. Firstly, the development of a multiplexed assay for screening the inhibitors of STATs-SH2 binding is novel. Secondly, it is applicable to most other binding assays. We chose two STAT proteins, STAT3 and STAT5b, which belong to the same protein family. However, this system can also be used for two different families of proteins, through peptide-protein, peptide-peptide, or protein-protein interactions. Thirdly, it can concurrently identify specific or selective hits, thereby simplifying the characterization of primary hits. The multiplexing strategy can significantly reduce the amount of compounds, time and human resources necessary for a screening campaign. In addition, either screening for target protein binding and counter screening for unrelated binding, or screening and selectivity screening for related binding, can be performed simultaneously in order to determine specificity or selectivity, respectively. We predict that specific inhibitors that are not false positives could be identified by only performing this multiplexed assay. We have demonstrated the use of the multiplexed assay to identify selective inhibitors.

However, there are limitations to this approach. The binding must be conducted under the same assay conditions. Both binding assays must use the same assay buffer, and be stable to DMSO and over the reaction time. We developed the STAT3 and STAT5b binding assays under the same assay conditions. Additionally, labeled peptides which do not cross-react with the counter proteins are also necessary. We used two labeled peptides, DIG-GpYLPQTV and FITC-GpYLVLDKW, for STAT3 and STAT5b binding in our multiplexed assay, respectively. These labeled peptides did not significantly cross-react with their counter proteins (Figure S7).

### Hit Identification and Initial SAR Studies using the Multiplexed Assay

**Figure 5:**
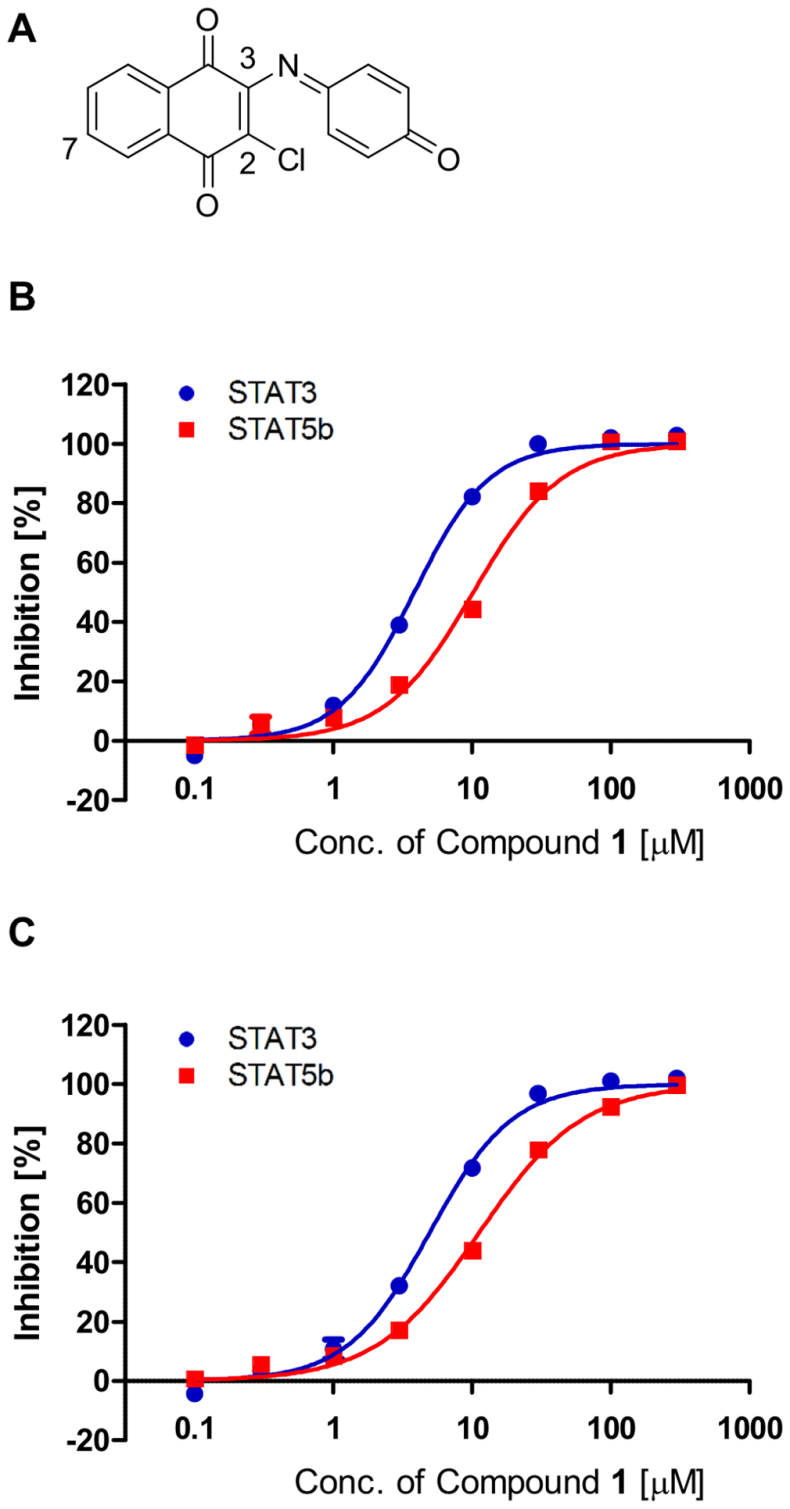
Identification of Compound 1 and its inhibition of STAT3- and STAT5b-SH2 binding. (A) Chemical structure of Compound **1**. (B) Dose-dependent inhibition of Compound **1** in the single assay. (C) Dose-dependent inhibition of Compound **1** in the multiplexed assay. Each point represents the mean from three replicates, and the error bars represent the standard deviation from the mean.

In order to identify a new chemotype for STAT3/STAT5b-SH2 antagonists, we performed a HTS campaign on our in-house compound libraries using the multiplexed assay. Several hit compounds were identified, including a 2-chloro-1,4-naphthalenedione derivative, Compound **1** (Figure 5A). Compound **1** inhibited both STAT3- and STAT5b-SH2 binding in a dose-dependent manner (Figures 5B, 5C). Compound **1** preferentially inhibited STAT3 over STAT5b. The IC_50_ values in the multiplexed assays were 5.0 µM and 11 µM for the STAT3- and STAT5b-SH2 binding, and those for the non-multiplexed single assays were 3.8 µM and 10 µM, respectively. The IC_50_ values were similar for the multiplexed and the single assays.

Compound **1** inhibited neither STAT1-SH2 nor Grb2-SH2 binding in the AlphaScreen assay (Figure S8A). In addition, the control signals generated by the biotinylated FITC or biotinylated DIG were not affected by this hit compound (Figures S8B, S8C). These results suggest that Compound **1** selectively inhibits STAT3/STAT5b over other SH2-containing proteins and preferentially inhibits STAT3 in comparison to STAT5b without influence to the Alpha assay system.

**Figure 6:**
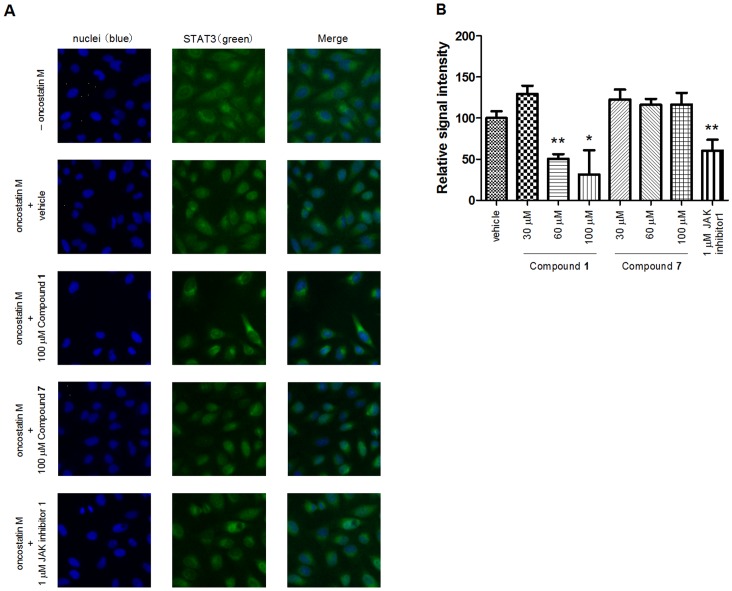
Inhibition of the STAT3 nuclear translocation by Compound 1 in HeLa cells. (A) Fluorescence images of the STAT3 proteins in the nuclei of HeLa cells. The HeLa cells were pretreated with or without the test compounds, and then either treated with oncostatin M or left untreated. The cell nuclei were stained with Hoechst 33342 and the STAT3 proteins were visualized with anti-STAT3 antibody. The merged image showed the fusion of blue (nuclei) and green (STAT3) fluorescence. The test compounds treated are shown in the figure. The cells treated with neither oncostatin M nor the test compounds are shown as “–oncostatin M”. (B) Dose-dependent inhibition of the STAT3 nuclear translocation by Compound **1**. The mean fluorescent values of STAT3 in the nuclei were calculated. The signals with oncostatin M but not the test compounds (“vehicle”) and “–oncostatin M” were used for the 100% and 0% signals, respectively. The relative signal intensity was calculated in each well. Each point represents the mean from three replicates, and the error bars represent the standard deviation from the mean (*P<0.05 and **P<0.01 versus vehicle in Student’s *t*-test). JAK inhibitor 1 was used as a positive control.

In order to analyze the biological activity of Compound **1** for the STAT3 protein, we investigated the nuclear translocation by immunestaining. The nuclear translocation of STAT3 protein was observed in HeLa cells by treating them with oncostatin M, which is a pleiotropic cytokine that belongs to the IL-6 family cytokines, although STAT3 protein was detected in the cytosol before oncostatin M was applied. Pretreatment of the cells with Compound **1** before oncostatin M stimulation reduced the STAT3 nuclear translocation in a dose-dependent manner (Figure 6, Figure S9). When the cells were pretreated with 60 and 100 µM Compound **1**, the fluorescent intensity of STAT3 in the nuclei was significantly reduced to 51% and 31% of the vehicle treatment, respectively. The inhibitory effect of 60 µM Compound **1** was comparable to that of 1 µM JAK inhibitor 1 (61%), which was used as a positive control. In addition, we investigated the effect of Compound **1** on STAT3 transcriptional activity by reporter gene assay. As a result, pretreatment of the cells with Compound **1** before oncostatin M stimulation reduced the STAT3 transcriptional activity in a dose-dependent manner (Figure S10). Therefore, it was confirmed that Compound **1** inhibited STAT3 activity in the STAT3-SH2 cell-free assay and also the nuclear translocation and transcriptional activation of STAT3 in cell-based assays. However, a ten-fold higher concentration of Compound **1** was required to suppress STAT3 nuclear translocation and transcriptional activity than in vitro STAT3-SH2 binding. Such differences between the in vitro and in vivo concentrations are similar to those of JAK inhibitor 1. IC_50_ values of JAK inhibitor 1 in the in vitro assay were reported to be 15 nM, 1 nM, 5 nM and 1 nM for JAK1, JAK2, JAK3 and TYK2, respectively [20]. However, more than a 100-fold higher concentration of JAK inhibitor 1 was needed to suppress STAT3 nuclear translocation and transcriptional activity in the cell-based assay. Presumably, physicochemical properties such as solubility, stability and cell-permeability may affect such discrepancies.

In order to investigate the SAR, we obtained commercially available 2-chloro-1,4-naphthalenedione derivatives and examined their inhibitory activity on the STAT3- and STAT5b-SH2 binding using the multiplexed assay (Table 2). The 3-substituent effect was observed on the activity and selectivity against STAT3 and STAT5b. In comparison with the 3-quinoneimine moiety (Compound **1**), the 3-anilino substituent (Compound **2**) exhibited approximately 3-fold and 8-fold increase in the inhibitory activity against STAT3 and STAT5b, respectively. Replacement of the 3-quinoneimine moiety with an alkyl chain (Compound **3** and **4**), a halogen (Compound **5**) or 4-methylpiperazin (Compound **7**) substituent decreased the inhibitory activity against both STAT3 and STAT5b. Interestingly, the introduction of 3-morpholino and 7-morpholinosulfonyl substituents (Compound **6**) maintained the inhibitory activity against not only STAT3 but also STAT5b. In consistent with the results from the multiplexed assay, Compound **7** did not inhibit the STAT3 nuclear translocation (Figure 6, Figure S9). Although these initial SAR studies are limited, they suggest that the 2-chloro-1,4-naphthalenedione ring with appropriate 3-substituents is important for STAT3 and STAT5b inhibitory activity. Compound **1**, a screening hit in this multiplexed assay system, could potentially be a useful tool for developing new anticancer agents based on selective STAT3/STAT5 inhibition. Further SAR studies of these agents and their related derivatives are underway to design more potent and selective STAT3/STAT5 inhibitors.

**Table 2:** Structure-activity relationships for the Compound 1 analogs for the STAT3- and STAT5b-SH2 bindings.

Compound	3-substituent	7-substituent	STAT3	STAT5b
**1**	quinoneimino	-H	5.0±0.073	11±0.27
**2**	2-ethoxyanilino	-H	1.5±0.052	1.4±0.019
**3**	2-[(2-hydroxyethyl)amino]ethylamino	-H	12±0.059	20±0.26
**4**	acetylamino	-H	23±0.68	>30
**5**	-Cl	-H	12±0.59	20±0.33
**6**	morpholino	morpholinosulfonyl	3.8±0.064	13±0.38
**7**	1-methylpiperazino	-H	>30	>30

The mean and the SEM of IC_50_ values (µM) of the tested compounds on STAT3- and STAT5b-SH2 bindings were shown in the table. These values were determined from three independent experiments in the multiplexed binding assay.

### Conclusion

We have successfully developed a multiplexed assay for two different types of STAT-SH2 binding by combining different colored AlphaLISA and AlphaScreen beads in a single well. We performed a HTS campaign with this assay system to identify STAT3- and STAT5b-SH2 binding inhibitors in our compound libraries. The compound Compound **1** inhibited both STAT3- and STAT5b-SH2 bindings *in vitro* and also the nuclear translocation and transactivation of STAT3 in cell-based assays. Initial SAR studies suggested its potential as a lead compound for developing selective STAT3/5b-SH2 antagonists. Therefore, our multiplexed assay is suitable for screening STATs inhibitors in primary HTS for identifying potential lead compounds and for the further structural optimization of STAT3/STAT5b inhibitors.

## Materials and Methods

### Reagents

Anti-STAT3 and anti-STAT5 antibodies were purchased from Cell Signaling Technologies. Horseradish peroxidase (HRP) conjugated anti-rabbit IgG were obtained from GE Healthcare Bio-Sciences. The digoxygenin (DIG)-labeled peptides, DIG-C2-GpYLPQTV and DIG-C6-GpYLPQTV, were synthesized by Sigma-Aldrich, and the 5-carboxyfluorescein (FITC)-labeled peptide, FITC-C6-GpYLVLDKW, was synthesized by Nippi Biomatrix Laboratory. Anti-DIG and anti-FITC acceptor beads and streptavidin-coated donor beads were purchased from PerkinElmer Life Sciences. *E. coli*, strain AVB99, was bought from Avidity, and *E. coli*, strain BL-21 Star (DE3) pLysS, was purchased from Life Technologies Corporation. pBluescript II KS (+) was obtained from Stratagene, and pET-28a (+) was bought from Novagene. STAT5 inhibitor (N′-((4-Oxo-4H-chromen-3-yl)methylene)nicotinohydrazide) was purchased from Merck Millipore. Oncostatin M was bought from Wako Pure Chemical Industries. The compounds Compound **1**, Compound **3**, Compound **4**, Compound **6** and Compound **7** were purchased from Enamine, and Compound **2** and Compound **5** were purchased from Maybridge. The other reagents were purchased from Nacalai Tesque.

### Cell Lines and Culture

HeLa cells were purchased from the American Type Culture Collection. The STAT3 reporter HeLa stable cell line for the Luciferase reporter gene assay was bought from Panomics. Cells were maintained in Dulbecco’s Modified Eagle’s Medium (DMEM) supplemented with 10% (v/v) fetal bovine serum, 20 U/mL penicillin, and 20 µg/mL streptomycin at 37°C in a humidified atmosphere containing 5% CO_2_.

### Plasmid Construction and Protein Expression

Full-length human STAT3 and STAT5b cDNA were obtained from a human cDNA library using PCR techniques and subcloned into pBluescript II KS (+). The corresponding cDNA regions of amino acids 136 to 705 of human STAT3 and 136 to 703 of human STAT5b were each amplified from the full length cDNA by PCR, and cloned into the *Hin*dIII/*Xho*I sites of modified pET-28a (+) carrying the Avi-tag and 6×His-tag at the N-terminus of the proteins [40]. *E. coli* BL-21 Star (DE3) pLysS transfected with the pBirAcm plasmid isolated from AVB99, and the pET-28a (+) plasmid, was grown at 37°C in LB medium containing 10 µg/mL chloramphenicol and 30 µg/mL kanamycin to an OD600 of 0.6. The cells were induced with 0.2 mM isopropyl-β-D-thiogalactopyranoside and 10 µM biotin for 3 h at 30°C and subsequently harvested by centrifugation. The bacterial pellet was suspended in lysis buffer (50 mM Tris-HCl (pH 7.4), 500 mM NaCl, 5 mM β-mercaptoethanol, 0.1% (w/v) Nonidet P-40 (NP-40), and 1 tablet of complete protease inhibitor cocktail tablets per 50 mL). The cells were lysed with 10 cycles of sonication, each consisting of a constant pulse for 1 min on ice. Cell lysates were centrifuged at 12,000 g for 20 min at 4°C, and the supernatant and the precipitate were the soluble and the insoluble fractions, respectively. The supernatant was loaded onto a HisTrap HP column (GE Healthcare Bio-Sciences) using an AKTA system (GE Healthcare Bio-Sciences). Proteins non-specifically bound to the column were washed out with a wash buffer (50 mM Tris-HCl (pH 7.4), 300 mM NaCl, 0.2 mM EDTA, 0.1% (w/v) NP-40, 5 mM β-mercaptoethanol, 20 mM imidazole, and 10% (v/v) glycerol). Recombinant proteins were eluted with elution buffer (50 mM Tris-HCl (pH 7.4), 300 mM NaCl, 0.2 mM EDTA, 0.1% (w/v) NP-40, 5 mM β-mercaptoethanol, 10% (v/v) glycerol, and 10–250 mM imidazole). Imidazole in the elution fraction was removed by dialysis against 10 mM HEPES (pH 7.4), 50 mM NaCl, 10 mM β-mercaptoethanol, 0.1 mM EGTA, 0.02% (w/v) NP-40, 0.2 mM phenylmethylsulfonyl fluoride, and 10% (v/v) glycerol at 4°C overnight. STAT1 and Grb2 protein were prepared as described in previous report [29]. The purified proteins were snap-frozen in liquid nitrogen and stored at –80°C before use.

### CBB Staining and Western Blotting

The proteins were subjected to SDS-PAGE, and were stained with Quick-CBB (Wako Pure Chemical Industries). For Western blotting, the proteins were transferred onto a nitrocellulose membrane. The membranes were blocked with 5% (w/v) skim milk in TBS (pH 7.4) and 0.1% (w/v) Tween 20 overnight at 4°C. They were then incubated with primary antibodies diluted at 1∶2,000 with 1.5% (w/v) skim milk in TBS (pH 7.4) and 0.1% (w/v) Tween 20 for 2 h at room temperature, followed by incubation with HRP-conjugated secondary antibodies diluted at 1∶10,000 in TBS (pH 7.4) and 0.1% (w/v) Tween 20 for 1 h at room temperature. Immunoblots were developed using chemiluminescent substrate.

### Alpha-based Binding Assays

Both AlphaLISA and AlphaScreen are bead-based nonradioactive binding assay systems for detecting biomolecular interactions in a microplate format. The binding of the biological partners brings the donor and acceptor beads into close proximity, and AlphaLISA produces a specific signal around 615 nm and AlphaScreen emits a broad signal between 520 and 620 nm. The assays were performed in a final reaction volume of 25 µL of the assay buffer, which contained 10 mM HEPES-NaOH (pH 7.4), 50 mM NaCl, 1 mM EDTA (pH 8.0), 0.1% (w/v) NP-40, and 10 ng/µL (w/v) BSA in a 96-well microplate at 25°C. The labeled phosphotyrosyl (pTyr, pY) peptide probes were DIG-GpYLPQTV and FITC-GpYLVLDKW for STAT3 and STAT5b, respectively. In the single assay for STAT3 binding, 100 nM STAT3(136–705) protein was incubated with a test compound dissolved in DMSO for 30 min, and 1.0 nM DIG-GpYLPQTV was added and the mixture was incubated for 90 min. Streptavidin-coated donor beads (0.25 µg/well) and anti-DIG AlphaLISA acceptor beads (0.25 µg/well) were added and incubated for 90 min before the signals were measured with EnVison Xcite (PerkinElmer Life Sciences). In the single assay for STAT5b binding, 20 nM STAT5b(136–703) was incubated with a test compound for 30 min, and 1.0 nM FITC-GpYLVLDKW was added and incubated for 90 min. Streptavidin-coated donor beads (0.25 µg/well) and anti-FITC AlphaScreen acceptor beads (0.25 µg/well) were added and incubated for 90 min before the signals were measured with EnVison Xcite. In the multiplexed assay, 100 nM STAT3(136–705) and 20 nM STAT5b(136–703) were incubated with a test compound for 30 min, and 1.0 nM DIG-GpYLPQTV and 1.0 nM FITC-GpYLVLDKW were added and incubated for 90 min. Streptavidin-coated donor beads (0.50 µg/well), anti-DIG AlphaLISA (0.25 µg/well), and anti-FITC AlphaScreen acceptor beads (0.25 µg/well) were added and incubated for 90 min before the signals were measured with EnVison Xcite. AlphaLISA and AlphaScreen signals for measuring STAT3- and STAT5b-SH2 binding, respectively, can be separately measured by using a dysprosium 572 nm and a europium 615 nm narrow bandwidth filter. In inhibitory assays, these signals for the bound state and the free state were represented as 0% and 100% inhibition, respectively. The bound state was measured by incubating the labeled peptide and the protein without test compounds. The free state was measured by incubating the same mixture with an additional 10 µM non-labeled peptide contained phosphorylated tyrosine, which competitively binds to its corresponding protein. The IC_50_ value was calculated by Graph pad prism 4.0. The mean and the standard error (SEM) of IC_50_ values were determined from three independent experiments. The Z’ value was calculated using the equation Z’ = 1–(3×SD_bound +3×SD_free)/(Signal_bound – Signal_free). STAT1 and Grb2 binding assays were performed as described in previous report [29]. In the assays for selectivity of Compound **1**, 100 pM biotinylated DIG or 100 pM biotinylated FITC was incubated with 10 µM or 30 µM Compound **1** for 30 min. Streptavidin-coated donor beads (0.25 µg/well) and anti-DIG AlphaLISA acceptor beads (0.25 µg/well) or anti-FITC AlphaScreen acceptor beads (0.25 µg/well) were added and incubated for 90 min before the signals were measured with EnVison Xcite.

### Nuclear Translocation Assay

The Cellomics STAT3 Activation Kit (Thermo Scientific) was used for the nuclear translocation assay. HeLa cells were incubated in a 96-well microplate for 24 h. Cells were pretreated with the test compounds for 1 hour, and 30 ng/mL (w/v) of oncostatin M was applied and the mixture was incubated for 10 min. The subsequent procedures were performed according to the manufacturer’s instructions. The cells were scanned with an ArrayScan VTI reader (Thermo Scientific), and analyzed with the Cytoplasm to Nucleus Translocation Application. The mean fluorescent values in the nuclei were calculated. The signals for vehicle treatment and no treatment (without oncostatin M) were represented as 100% and 0%, respectively, and the relative signal intensity was also calculated.

### Luciferase Reporter Gene Assay

STAT3 reporter HeLa stable cell lines were incubated in a 96-well microplate for 24 hours. Cells were pretreated with test compounds for 1 hour, and 10 ng/ml (w/v) of oncostatin M were applied and incubated for 4 hours. Cells were washed with medium not supplemented with phenol red, and Steady-Glo® reagent (Promega) was applied. After 5 min incubation, the signals were detected by ARVO Light 1420 (PerkinElmer Life Sciences). The relative signal intensity was calculated in each well as the ratio for the mean signal of vehicle.

### Statistical Analysis

In the nuclear translocation and the luciferase reporter gene assays, the statistical significance was calculated by Student’s *t*-test with *P<0.05 and **P<0.01 versus vehicle treatment.

## Supporting Information

Figure S1 **Dose dependence of the labeled peptides in each single assay.** (A) Various concentrations of DIG-C6-GpYLPQTV were used in the STAT3-SH2 AlphaLISA binding assay. (B) Various concentrations of FITC-C6-GpYLVLDKW peptide were used in the STAT5b-SH2 AlphaScreen binding assay. Each point is the mean from three replicates, and the error bars represent the standard deviation from the mean. The signals for 1.0 nM DIG-C6-GpYLPQTV with 100 nM STAT3(136–705) (A) or 1.25 nM FITC-C6-GpYLVLDKW with 20 nM STAT5b(136–703) (B) represent a value of 100%.
(TIF)

Figure S2 **Inhibition of STAT3-SH2 binding by DIG-C2-GpYLPQTV.** DIG-C2-GpYLPQTV was used as a competitor in the binding assay for STAT3(136–705) protein and FITC-C6-GpYLPQTV peptide in the single assay. Each point is the mean from three replicates, and the error bars represent the standard deviation from the mean.
(TIF)

Figure S3 **Effect of dimethylsulfoxide on the single assays.** (A) STAT3(136–705) binding by AlphaLISA. (B) STAT5b(136–703) binding by AlphaScreen. The DMSO concentration shown in this figure was contained with the reactant. Each point represents the mean from three replicates, and the error bars represent the standard deviation from the mean. The signals for 100 nM STAT3(136–705) in 1.0% DMSO (A) or 20 nM STAT5b(136–703) on 1.0% DMSO (B) represent a value of 100%.
(TIF)

Figure S4 **Effect of the reaction time on the single assays.** (A) STAT3(136–705) binding by AlphaLISA. (B) STAT5b(136–703) binding by AlphaScreen. The reaction time for the labeled peptides and STAT proteins is shown. Each point represents the mean from three replicates, and the error bars represent the standard deviation from the mean. The signals for 100 nM STAT3(136–705) after 90 min (A) or 20 nM STAT5b(136–703) after 90 min (B) a value of 100%.
(TIF)

Figure S5 **Effect of sodium chloride (NaCl) in the single assays.** (A) STAT3(136–705) binding by AlphaLISA. (B) STAT5b(136–703) binding by AlphaScreen. The NaCl concentration shown in this figure was contained in the reactant. Each point represents the mean from three replicates, and the error bars represent the standard deviation from the mean. The signals for 100 nM STAT3(136–705) in 50 mM NaCl (A) or 20 nM STAT5b(136–703) in 50 mM NaCl (B) represent a value of 100%.
(TIF)

Figure S6 **Selective inhibitory effect of Stattic and STAT5 inhibitor in the multiplexed assay.** (A) Dose-dependent inhibitory effect of Stattic. (B) Dose-dependent inhibitory effect of STAT5 inhibitor. The STAT3- and STAT5b-SH2 binding was detected by AlphaLISA and AlphaScreen, respectively, in the multiplexed assay. Each point represents the mean from three replicates, and the error bars represent the standard deviation from the mean.
(TIF)

Figure S7 **Cross-reactivity between AlphaLISA STAT3 and AlphaScreen STAT5b binding in the multiplexed assay.** (A) DIG-GpYLPQTV peptide-dose dependence on STAT3 and STAT5b. Various concentrations of DIG-GpYLPQTV, 1.0 nM FITC-GpYLVLDKW, 100 nM STAT3(136–705), and 20 nM STAT5b(136–703) were mixed in the same well, and both the AlphaLISA and AlphaScreen signals were measured in the multiplexed assay. (B) FITC-GpYLVLDKW peptide dose dependence on STAT3 and STAT5b binding. Various concentrations of FITC-GpYLVLDKW, 1.0 nM DIG–GpYLPQTV, 100 nM STAT3(136–705), and 20 nM STAT5b(136–703) were mixed in the same well, and both the AlphaLISA and AlphaScreen signals were measured in the multiplexed assay. The blue and the red spots indicate the AlphaLISA and AlphaScreen signals, respectively. Each point represents the mean from three replicates, and the error bars represent the standard deviation from the mean. The signals for 1.0 nM DIG-GpYLPQTV (A) and 1.0 nM FITC-GpYLVLDKW (B) represent a value of 100%.
(TIF)

Figure S8 **Selectivity of Compound** 1**.** (A) Effect of Compound **1** on the STAT1- and Grb2-SH2 binding in the AlphaScreen assay. Each point represents the mean from three replicates, and the error bars represent the standard deviation from the mean. (B) Effect of Compound **1** on the signal intensity generated by the combination of biotinylated DIG and AlphaLISA beads. (C) Effect of Compound **1** on the signal intensity generated by the combination of biotinylated FITC and AlphaScreen beads. The signals for vehicle and blank represent values of 100% and 0%, respectively.
(TIF)

Figure S9 **Inhibitory effect of the STAT3 nuclear translocation by Compound** 1 **and** 7 **in HeLa cells.** The fluorescent intensities of the STAT3 in the nuclei of the individual cells were calculated, and the frequency was plotted in a histogram. JAK inhibitor 1 was used as a positive control. The cells treated with neither oncostatin M nor test compounds are labeled “–oncostatin M”.
(TIF)

Figure S10 **Dose-dependent inhibition of the STAT3 transcriptional activation by Compound** 1 **in HeLa cells.** The signals with oncostatin M but not Compound **1** was denoted as “vehicle”. The relative signal intensity was calculated in each well as the ratio for the mean signal of vehicle. Each point represents the mean from three replicates, and the error bars represent the standard deviation from the mean (*P<0.05 and **P<0.01 versus vehicle in Student’s *t*-test). JAK inhibitor 1 was used as a positive control.
(TIF)
