# Supplementary figures and images for: Novel Multiplexed Assay for Identifying SH2 Domain Antagonists of STAT Family Proteins
Source: PLoS One. 2013 Aug 16;8(8):e71646. doi: 10.1371/journal.pone.0071646 (PMC3745430; doi:10.1371/journal.pone.0071646)

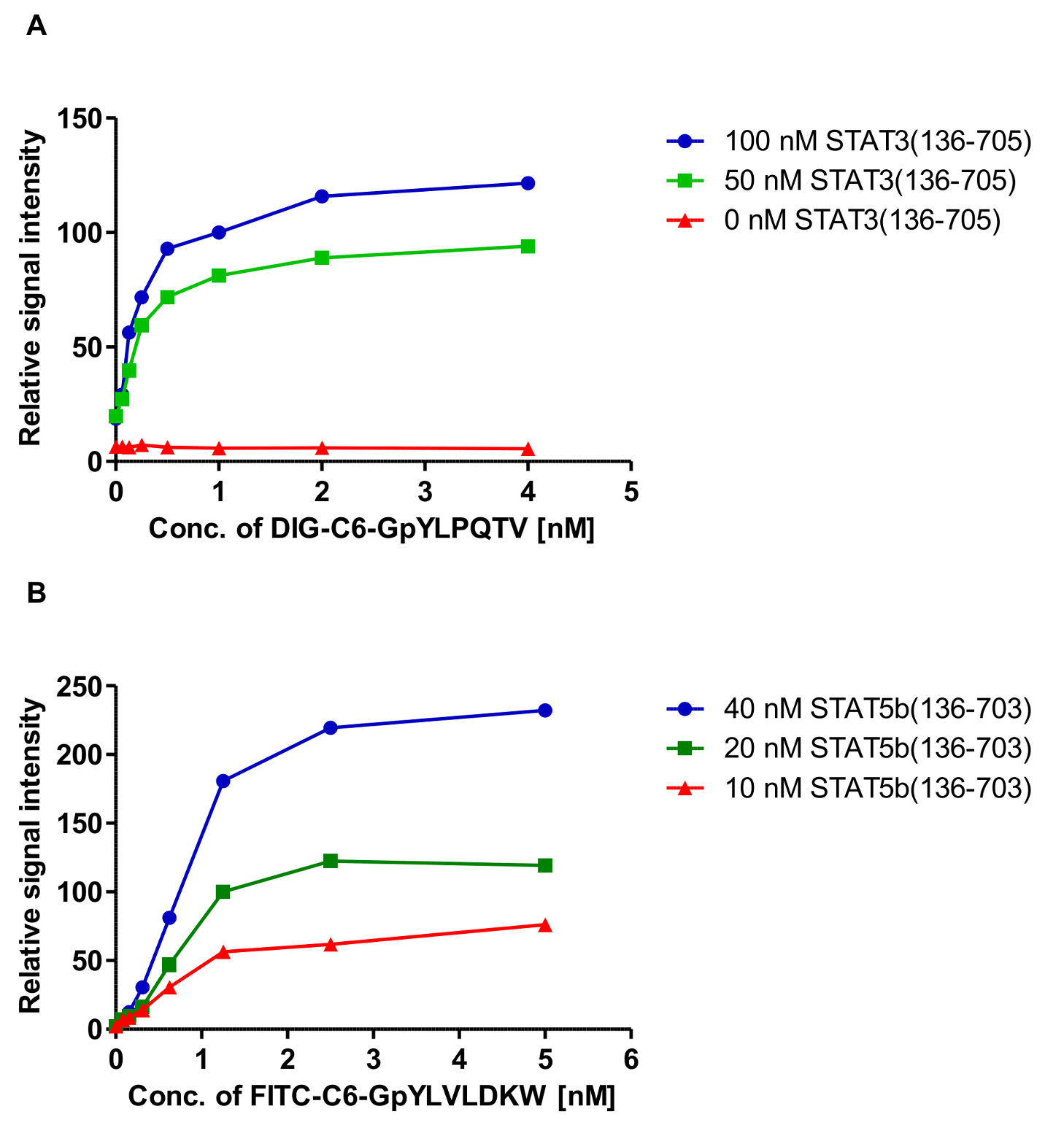

Supplement: Figure S1 — Dose dependence of the labeled peptides in each single assay. (A) Various concentrations of DIG-C6-GpYLPQTV were used in the STAT3-SH2 AlphaLISA binding assay. (B) Various concentrations of FITC-C6-GpYLVLDKW peptide were used in the STAT5b-SH2 AlphaScreen binding assay. Each point is the mean from three replicates, and the error bars represent the standard deviation from the mean. The signals for 1.0 nM DIG-C6-GpYLPQTV with 100 nM STAT3(136–705) (A) or 1.25 nM FITC-C6-GpYLVLDKW with 20 nM STAT5b(136–703) (B) represent a value of 100%. (TIF) [file pone.0071646.s001.tif]

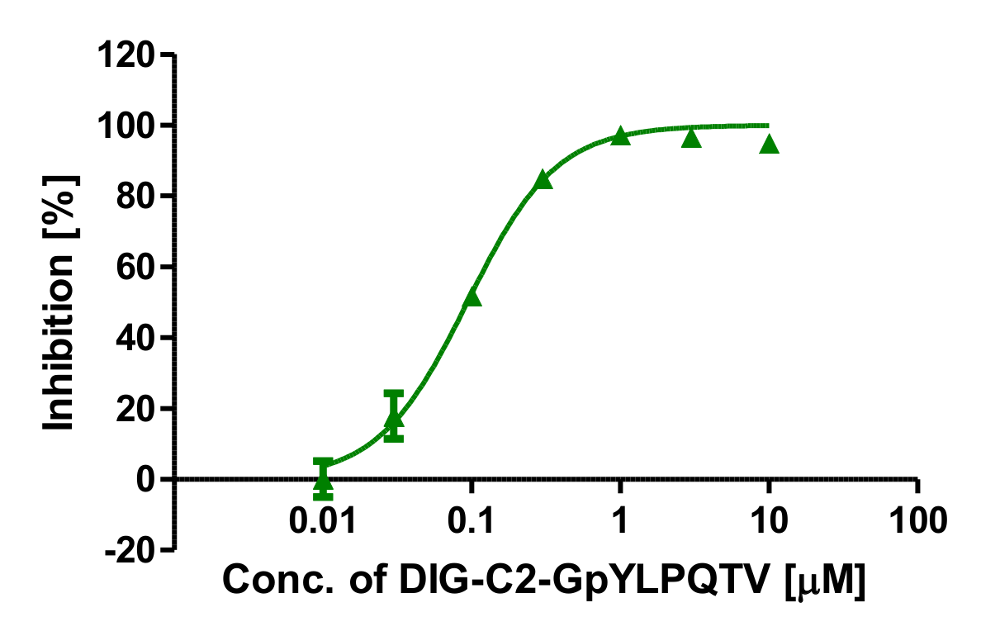

Supplement: Figure S2 — Inhibition of STAT3-SH2 binding by DIG-C2-GpYLPQTV. DIG-C2-GpYLPQTV was used as a competitor in the binding assay for STAT3(136–705) protein and FITC-C6-GpYLPQTV peptide in the single assay. Each point is the mean from three replicates, and the error bars represent the standard deviation from the mean. (TIF) [file pone.0071646.s002.tif]

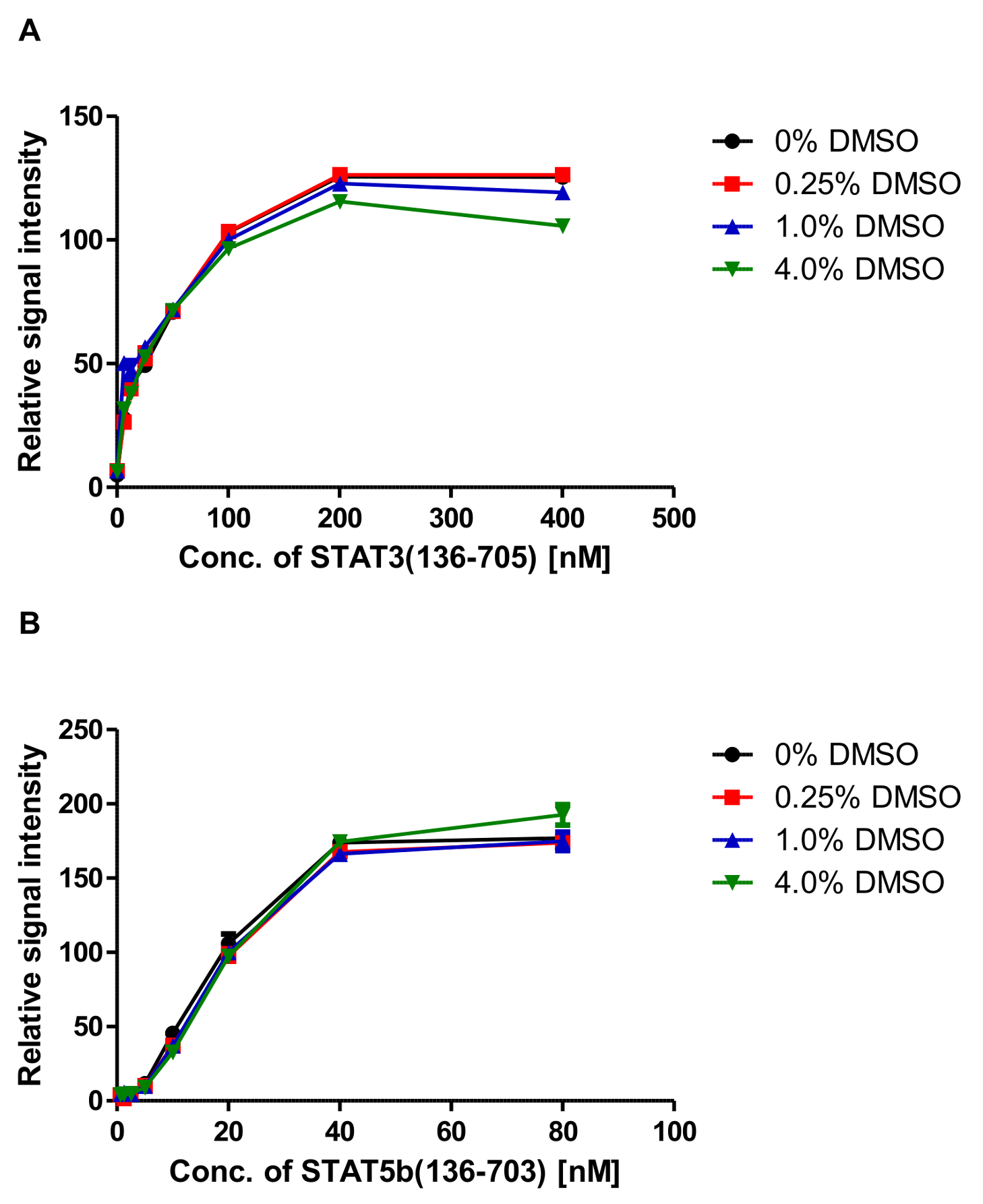

Supplement: Figure S3 — Effect of dimethylsulfoxide on the single assays. (A) STAT3(136–705) binding by AlphaLISA. (B) STAT5b(136–703) binding by AlphaScreen. The DMSO concentration shown in this figure was contained with the reactant. Each point represents the mean from three replicates, and the error bars represent the standard deviation from the mean. The signals for 100 nM STAT3(136–705) in 1.0% DMSO (A) or 20 nM STAT5b(136–703) on 1.0% DMSO (B) represent a value of 100%. (TIF) [file pone.0071646.s003.tif]

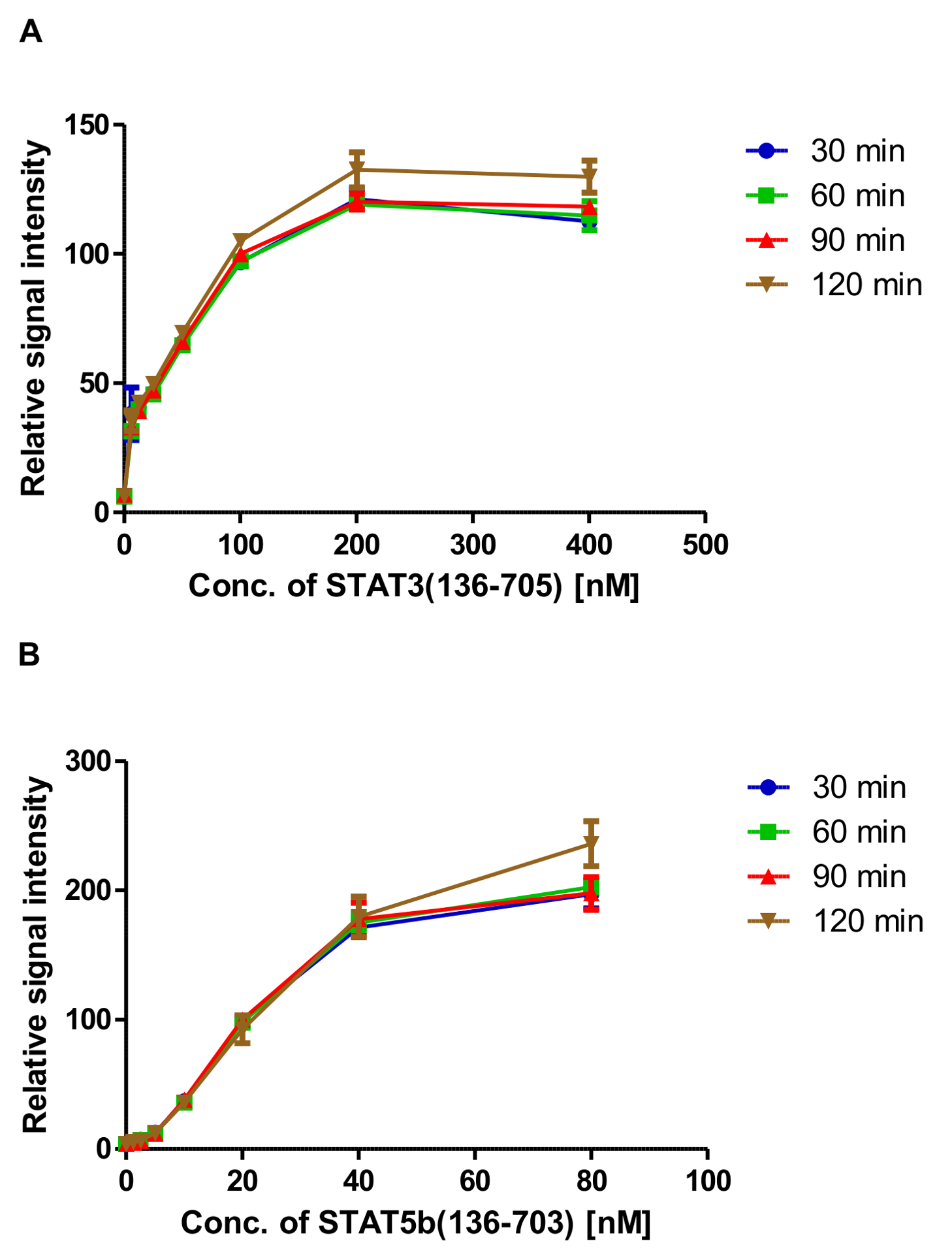

Supplement: Figure S4 — Effect of the reaction time on the single assays. (A) STAT3(136–705) binding by AlphaLISA. (B) STAT5b(136–703) binding by AlphaScreen. The reaction time for the labeled peptides and STAT proteins is shown. Each point represents the mean from three replicates, and the error bars represent the standard deviation from the mean. The signals for 100 nM STAT3(136–705) after 90 min (A) or 20 nM STAT5b(136–703) after 90 min (B) a value of 100%. (TIF) [file pone.0071646.s004.tif]

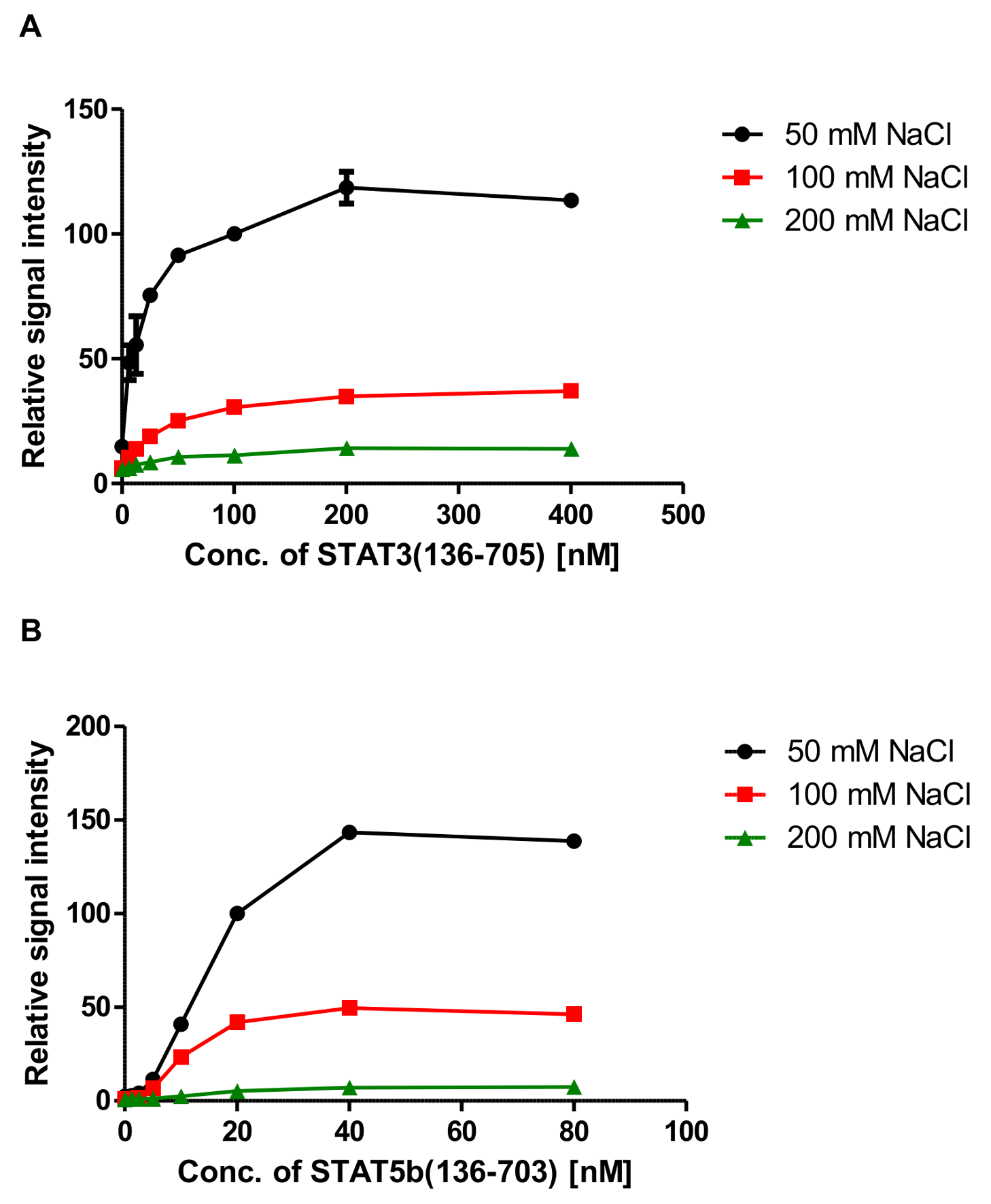

Supplement: Figure S5 — Effect of sodium chloride (NaCl) in the single assays. (A) STAT3(136–705) binding by AlphaLISA. (B) STAT5b(136–703) binding by AlphaScreen. The NaCl concentration shown in this figure was contained in the reactant. Each point represents the mean from three replicates, and the error bars represent the standard deviation from the mean. The signals for 100 nM STAT3(136–705) in 50 mM NaCl (A) or 20 nM STAT5b(136–703) in 50 mM NaCl (B) represent a value of 100%. (TIF) [file pone.0071646.s005.tif]

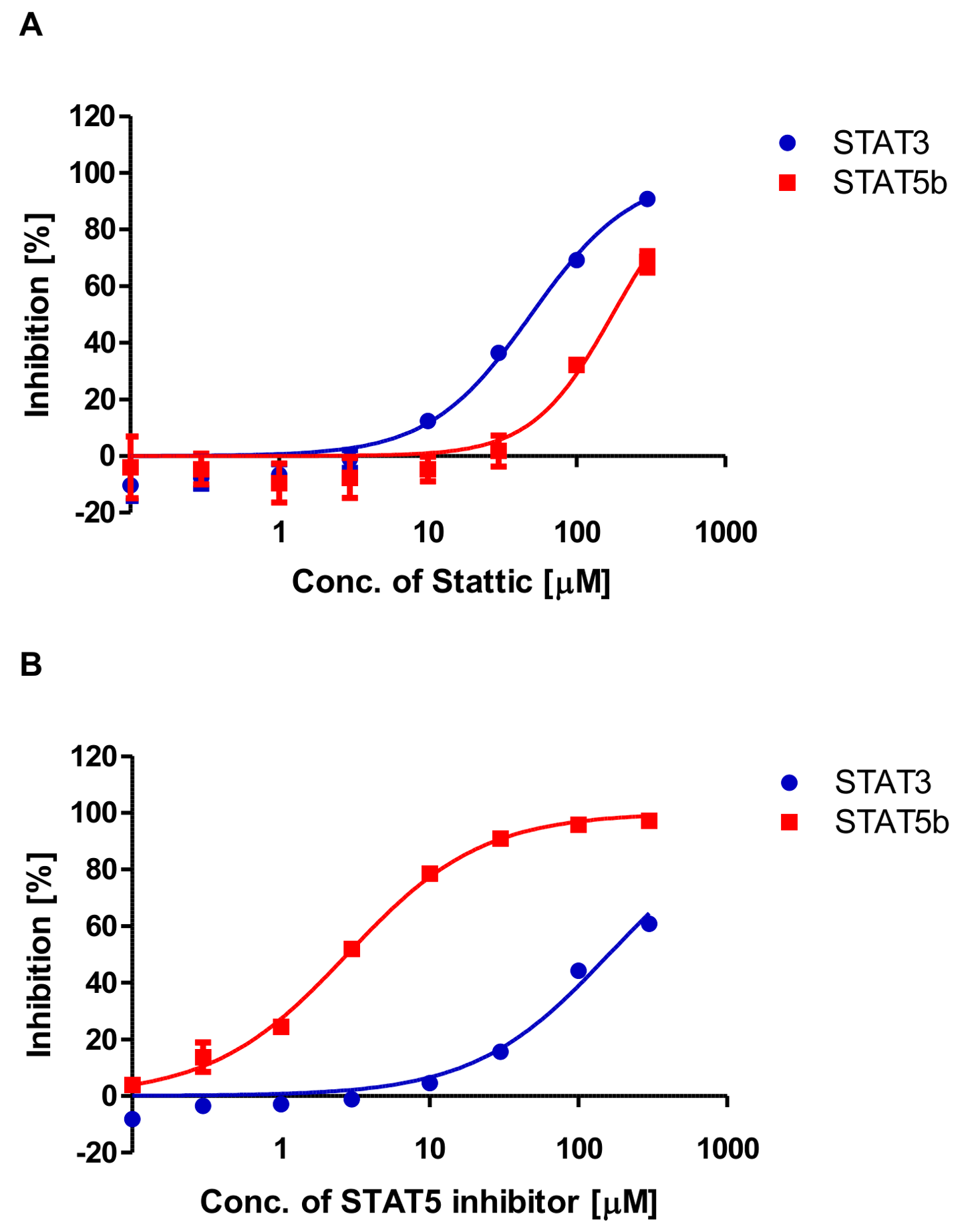

Supplement: Figure S6 — Selective inhibitory effect of Stattic and STAT5 inhibitor in the multiplexed assay. (A) Dose-dependent inhibitory effect of Stattic. (B) Dose-dependent inhibitory effect of STAT5 inhibitor. The STAT3- and STAT5b-SH2 binding was detected by AlphaLISA and AlphaScreen, respectively, in the multiplexed assay. Each point represents the mean from three replicates, and the error bars represent the standard deviation from the mean. (TIF) [file pone.0071646.s006.tif]

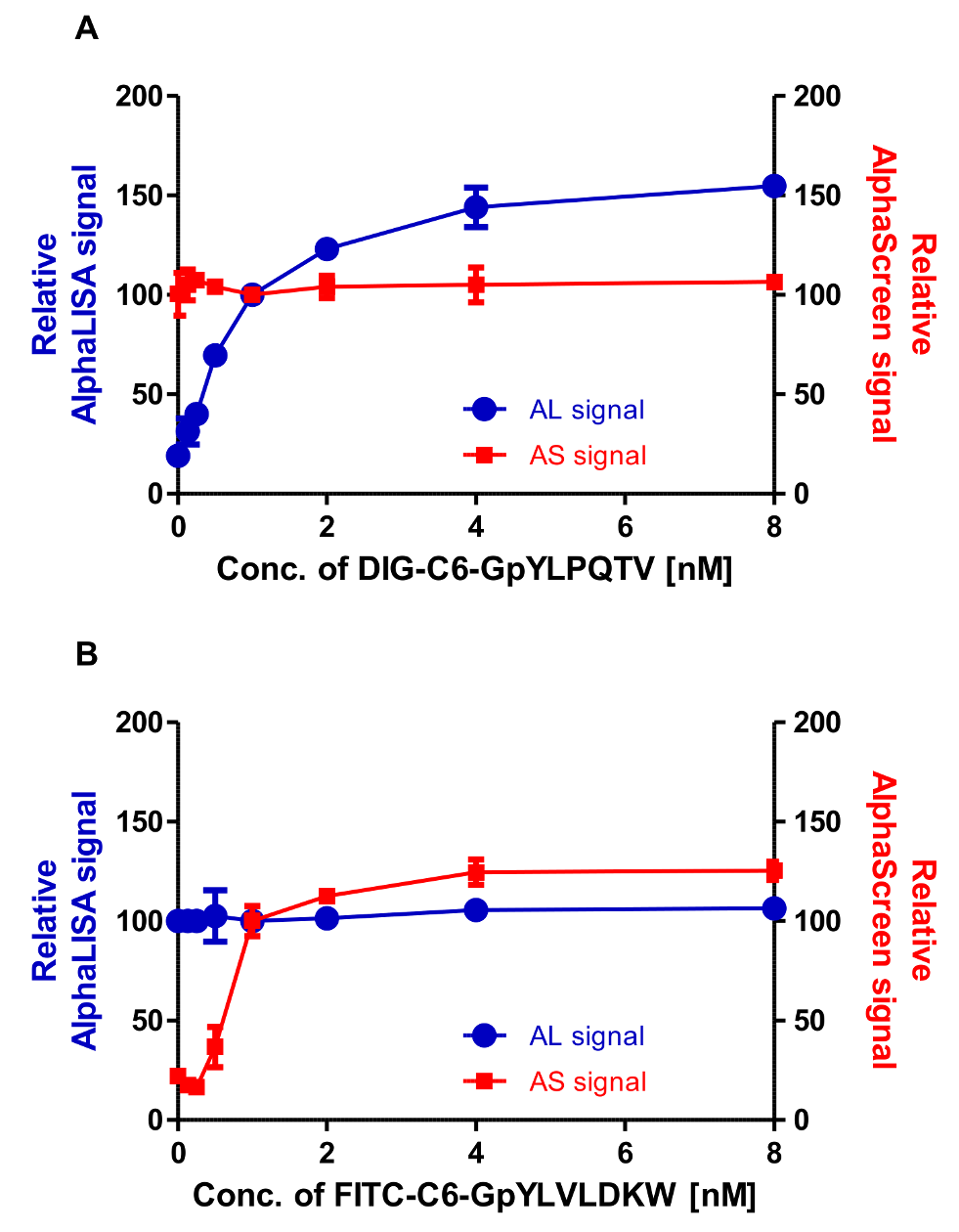

Supplement: Figure S7 — Cross-reactivity between AlphaLISA STAT3 and AlphaScreen STAT5b binding in the multiplexed assay. (A) DIG-GpYLPQTV peptide-dose dependence on STAT3 and STAT5b. Various concentrations of DIG-GpYLPQTV, 1.0 nM FITC-GpYLVLDKW, 100 nM STAT3(136–705), and 20 nM STAT5b(136–703) were mixed in the same well, and both the AlphaLISA and AlphaScreen signals were measured in the multiplexed assay. (B) FITC-GpYLVLDKW peptide dose dependence on STAT3 and STAT5b binding. Various concentrations of FITC-GpYLVLDKW, 1.0 nM DIG–GpYLPQTV, 100 nM STAT3(136–705), and 20 nM STAT5b(136–703) were mixed in the same well, and both the AlphaLISA and AlphaScreen signals were measured in the multiplexed assay. The blue and the red spots indicate the AlphaLISA and AlphaScreen signals, respectively. Each point represents the mean from three replicates, and the error bars represent the standard deviation from the mean. The signals for 1.0 nM DIG-GpYLPQTV (A) and 1.0 nM FITC-GpYLVLDKW (B) represent a value of 100%. (TIF) [file pone.0071646.s007.tif]

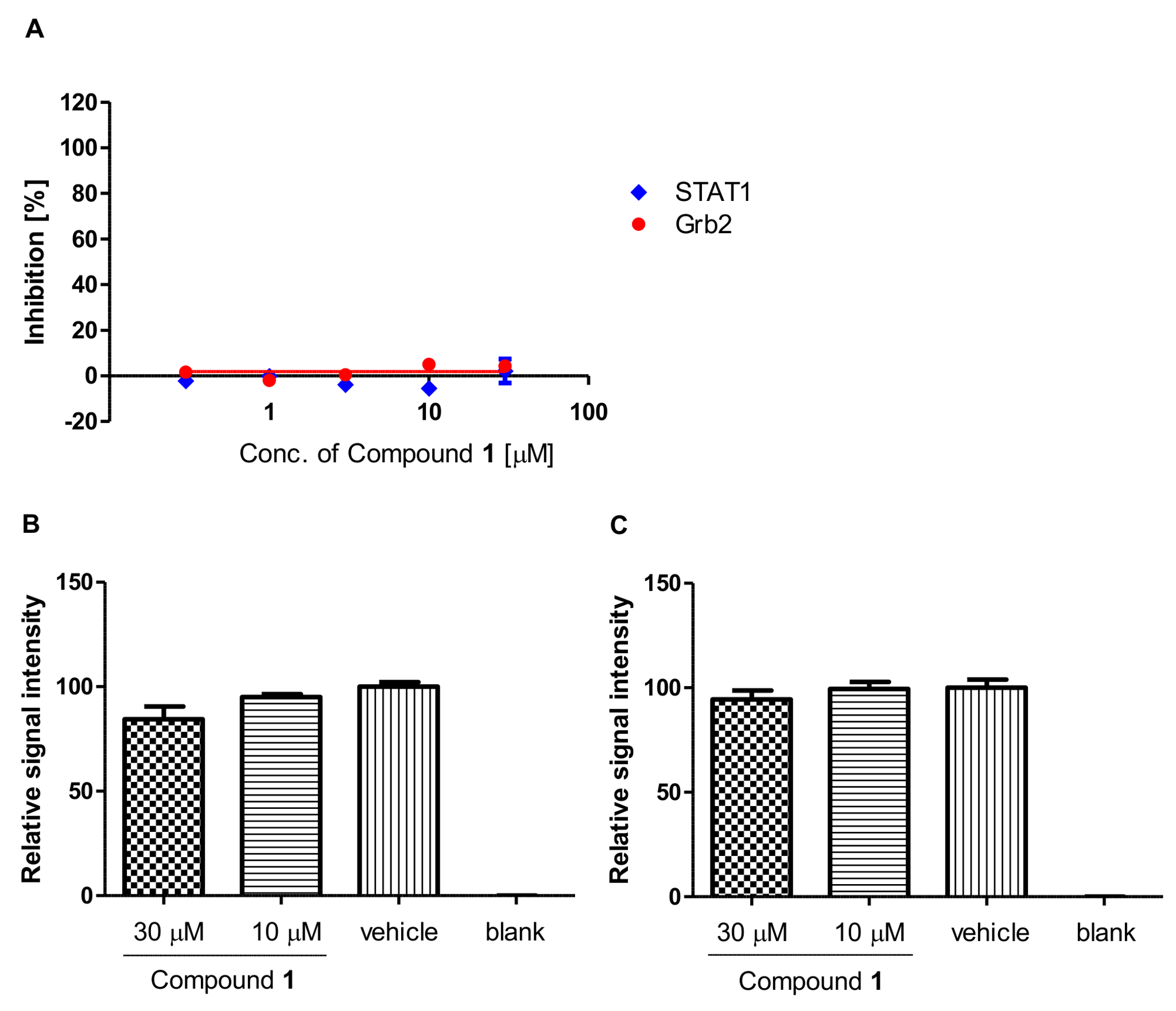

Supplement: Figure S8 — Selectivity of Compound 1. (A) Effect of Compound 1 on the STAT1- and Grb2-SH2 binding in the AlphaScreen assay. Each point represents the mean from three replicates, and the error bars represent the standard deviation from the mean. (B) Effect of Compound 1 on the signal intensity generated by the combination of biotinylated DIG and AlphaLISA beads. (C) Effect of Compound 1 on the signal intensity generated by the combination of biotinylated FITC and AlphaScreen beads. The signals for vehicle and blank represent values of 100% and 0%, respectively. (TIF) [file pone.0071646.s008.tif]

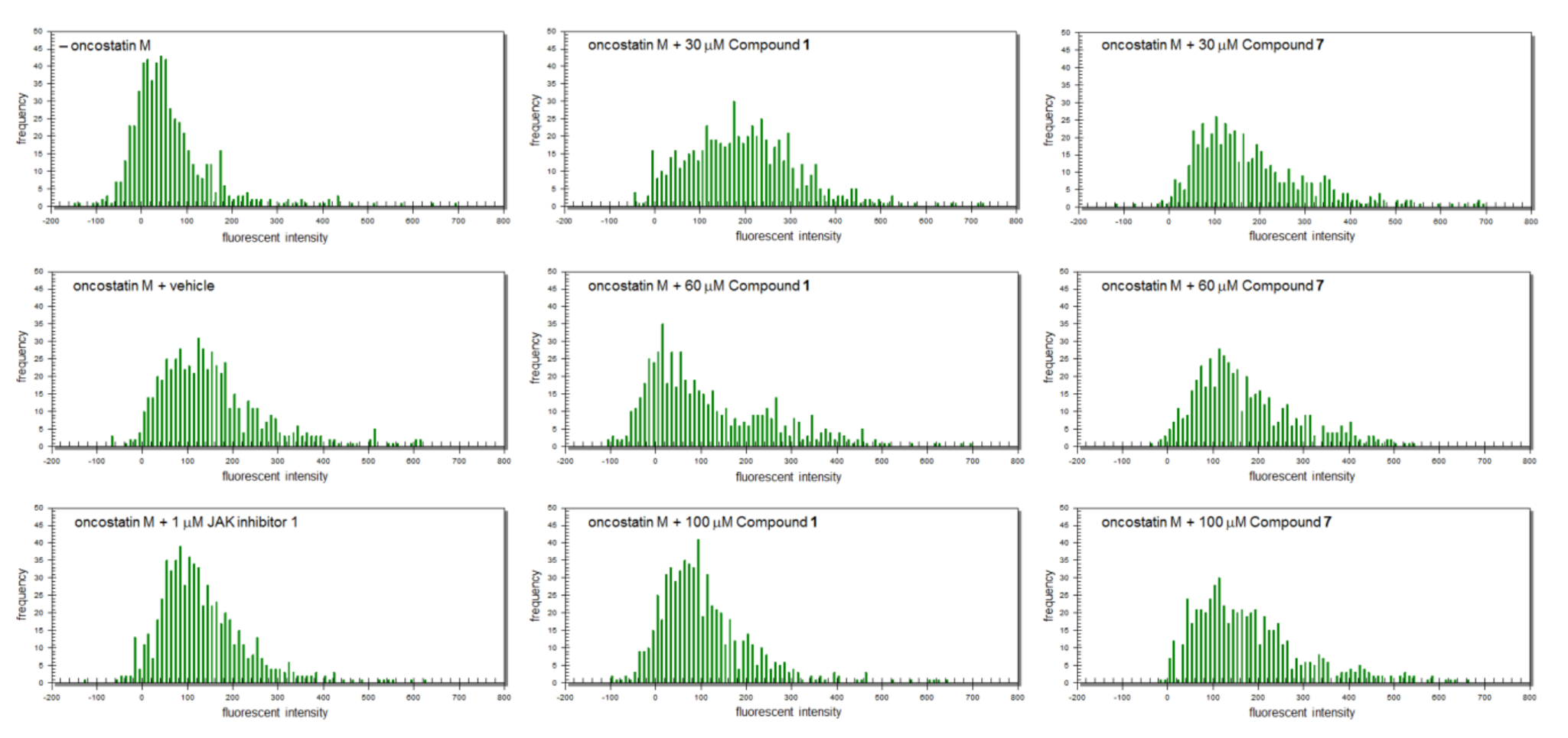

Supplement: Figure S9 — Inhibitory effect of the STAT3 nuclear translocation by Compound 1 and 7 in HeLa cells. The fluorescent intensities of the STAT3 in the nuclei of the individual cells were calculated, and the frequency was plotted in a histogram. JAK inhibitor 1 was used as a positive control. The cells treated with neither oncostatin M nor test compounds are labeled “–oncostatin M”. (TIF) [file pone.0071646.s009.tif]
